# Oral Administration to Nursing Women of *Lactobacillus fermentum* CECT5716 Prevents Lactational Mastitis Development: A Randomized Controlled Trial

**DOI:** 10.1089/bfm.2016.0173

**Published:** 2017-05-01

**Authors:** José A. Hurtado, Jose A. Maldonado-Lobón, M. Paz Díaz-Ropero, Katherine Flores-Rojas, José Uberos, José L. Leante, Laura Affumicato, María Luz Couce, José M. Garrido, Mónica Olivares, Juristo Fonollá

**Affiliations:** ^1^ Department of Neonatology, Hospital Materno-Infantil del CHU, Granada, Spain.; ^2^ Research Department, Biosearch Life, Granada, Spain.; ^3^ Department of Metabolism and Pediatrics Research, Hospital Reina Sofía, Córdoba, Spain.; ^4^ Department of Pediatrics, School of Medicine, University of Granada, Spain.; ^5^ Department of Neonatology, Hospital G.U. Santa Lucía, Cartagena, Murcia, Spain.; ^6^ Department of Neonatology, Hospital R.U., Málaga, Spain.; ^7^ Department of Neonatology, Hospital C.H.U., Santiago de Compostela, La Coruña, Spain.; ^8^ Department of Pediatrics, Hospital U., Salamanca, Spain.

**Keywords:** mastitis, prevention, probiotics

## Abstract

***Objective:*** The objective of this study is to evaluate the preventive effect of oral administration of *Lactobacillus fermentum* CECT5716 on mastitis incidence in lactating women.

***Methods:*** A randomized double-blinded controlled trial that included 625 women was conducted. Women who received preventive dose of antibiotic in the context of delivery were recruited 1–6 days after childbirth and randomly assigned to a group. Probiotic group received 1 capsule/day containing *L. fermentum* 3 × 10^9^ CFU, control group received 1 placebo capsule/day containing maltodextrin. The intervention period was 16 weeks. The primary outcome of the study was the incidence of clinical mastitis defined as at least two out of the three breast symptoms (pain, redness, and lump) and at least one of fever or flu-like symptoms (shivering, hot sweats, or aches).

***Results:*** Two hundred ninety-one women completed 16 weeks of treatment. Sixteen women in the probiotic group developed mastitis versus 30 women in the control group (odds ratio = 0.531; *p* = 0.058). Incidence rate of mastitis in the probiotic group was significantly lower than that in the control group (IR = 0.130 in the probiotic group versus IR = 0.263 in the control group; *p* = 0.021). Therefore, the oral administration of *L. fermentum* CECT5716 during lactation decreased by 51% the incidence rate of clinical mastitis. *Staphylococcus* spp. load at the end of intervention was significantly lower in breast milk of women in the probiotic group than in breast milk of women in the control group (*p* = 0.025).

***Conclusion:*** Consumption of the probiotic strain *L. fermentum* CECT5716 might be used during breastfeeding as an efficient strategy to prevent development of lactational mastitis in women.

Trial registration: NCT02203877.

## Introduction

Human microbiota plays an important and increasingly recognized role in human health. Dysbiosis in microbiota of different locations of human organism has been related to diseases such as inflammatory bowel disease, diabetes, obesity, allergy, vaginal infections, atopic dermatitis, and tooth decay.^1^ Interestingly, probiotics are showing beneficial effects on these diseases by restoring the balance of the microbiota.^2,3^ In 2003,the presence of a physiological microbiota was first described in human milk.^4,5^ Recent studies have revealed that dysbiosis in this specific microbiota is related to mastitis.^6–8^

Mastitis is an inflammatory condition of the breast usually associated with lactation. Dysbiosis in mastitis is characterized by proliferation of certain bacterial species such as *Staphylococcus* spp., which has been identified as one of the main bacterial groups related to mastitis.^6,9–11^ Data about the incidence of this problem are very variable probably because of differences in diagnosis criteria. WHO refers data of incidence highly variable that can reach 33%.^9^ During the past decade, different studies have demonstrated the capability of certain probiotic strains to balance the microbiota in human milk by reducing load of bacterial groups related to mastitis.^12–14^ The decrease in bacterial load was related to a decrease in the severity of the disease. These studies have provided some evidence about the potential of probiotic bacteria to efficiently treat the problem of mastitis.

*Lactobacillus fermentum* CECT5716 is a probiotic strain isolated from breast milk of healthy woman.^15^ This strain has shown powerful anti-infectious activity probably related to its antibacterial activity and inmuno-enhancing activity.^16–19^ Regarding mastitis, two different studies have demonstrated that *L. fermentum* CECT5716 significantly improves mastitis condition by decreasing *Staphylococcus* spp. load in breast milk.^13,14^ *Staphylococcus* spp. is the main causal agent of mastitis and it is a risk factor for the disease.^6,9,10^ The capability of *L. fermentum* CECT5716 to reduce this important risk factor for mastitis encourages us to perform a clinical trial to evaluate the potential of this strain to prevent the development of lactational mastitis.

## Materials and Methods

### Study design and protocol

A randomized double-blinded placebo-controlled multicenter trial was performed. The inclusion criteria were as follows: healthy women between 18 and 45 years with development of normal pregnancy, childbirth took place 1–6 days before recruitment, birth between 37 and 42 weeks of gestation, women who had received preventive antibiotic treatment between 48 hours before and 48 hours after childbirth (one dose was sufficient for inclusion regardless of the type of antibiotic), and women with firm intention to breastfeed their children for at least 16 weeks. Exclusion criteria during the study were mammary pathologies or children's pathologies that hinder or preclude breastfeeding and low expectation of adherence to the study protocol. Written informed consent was obtained from the women.

Sample size was estimated based on the effect on the main outcome of the study and the incidence of mastitis. Based on previous data about mastitis incidence, the study was designed to exhibit sufficient power (80%) to detect a difference between groups of 40% in the incidence rate of mastitis after treatment with a 0.05 significance level (Software R version 2.12.2). The number of women necessary was 258 per group, total sample size was increased up to 625 women to compensate dropouts.

Women were recruited and distributed into two study groups, according to a randomization generated by a computer program (R version 2.12.2). Probiotic group received *L. fermentum* CECT5716 for 16 weeks at doses of 3 × 10^9^ CFU/day. Control group received a placebo of maltodextrin.

*L. fermentum* CECT5716 was provided by Biosearch Life. Capsules containing 3 × 10^9^ CFU/capsule of *L. fermentum* CECT5716 or maltodextrin were prepared by Biofabri S.L. (A Relva s/n 36400 O Porriño, Pontevedra, Spain).

Women consumed one capsule per day containing probiotic (probiotic group) or maltodextrin (control group). Probiotic and placebo were provided in identical gelatin capsules packaged in identical plastic tubes labeled in plain white with a code number that referred to the manufacturing batch. As stability of probiotic bacteria is dependent on temperature of conservation, capsules were kept at 4°C throughout the study. The concentration of viable *Lactobacillus* species in the probiotic capsules was stable throughout the study (2.9 × 10^9^ CFU/capsule at the end of the intervention).

Twelve hospitals from different regions of Spain collaborated in the study. The study was performed in accordance with the Helsinki declaration, and the protocol was approved by the Regional Ethics Committee of the Sistema Andaluz de Salud based in Seville (Spain) for hospital in Andalusia region and for local Ethics Committees for the rest of hospitals. The trial was registered in the U.S. Library of Medicine (www.clinicaltrial.gov) with the number NCT02203877.

### Study outcomes

The primary outcome of the trial was the incidence rate of mastitis during the first 4 months of breastfeeding. Mastitis was defined as reported by Amir et al.^20^: at least two out of the three breast symptoms (pain, redness, and lump) and at least one of fever or flu-like symptoms (shivering, hot sweats, or aches).

Secondary outcomes were microbiota of breast milk at the end of intervention and in mastitis events, monthly questionnaire on evaluation of breast pain, and inflammatory markers in breast milk at the end of intervention and in mastitis events.

### Data collection

For mastitis diagnosis, the presence of local symptoms in breast (pain, redness, and lump) and systemic symptoms (fever, shivering, hot sweats, or aches) was recorded. Women were contacted monthly by phone and asked about mastitis symptoms and asked to score their breast pain from 0 (no pain) to 10 (extremely painful). In case of mastitis symptoms, diagnosis was confirmed by a corresponding medical doctor or midwife. Data about use of antibiotic, analgesic, and topic treatment for nipple and for breast symptoms were also collected.

As changes in diet might influence the results on main outcomes of the study, this variable was controlled by a Food Frequency Questionnaire. The questionnaire was completed by the women at the beginning and at the end of the intervention. Compliance of women was evaluated by collecting the remaining capsules at the end of intervention.

Breast milk samples were collected at the beginning and at the end of intervention. In case of mastitis event, breast milk samples were also collected. For breast milk sample collection, nipple and mammary areola were cleaned with soap and water and an antibacterial (chlorhexidine) solution was applied. Breast milk samples were obtained by manual expression and collected in sterile tubes after discarding the first drops of milk. Samples were preserved at −20°C and processed within 1 month.

### Breast milk bacteria quantification

To estimate the concentration of total bacteria in breast milk, appropriate dilutions of samples in buffered peptonized water (bioMérieux SA, Marcy de Marcy l'Etoile, France) were spread in quadruplicate onto plates of plate count agar (PCA) and Wilkins-Chalgren anaerobe agar (WCA) (Oxoid, Basingstoke, United Kingdom). The cultures were incubated in aerobic (PCA) and anaerobic (WCA) conditions (AnaeroGen; Oxoid, Basingstoke, United Kingdom) at 37°C for 48 hours. After the incubation, the colonies grown on the culture media were counted, and the number of viable microorganisms per milliliter of milk (CFU/mL) was calculated.

*Staphylococci*, *Streptococci*, and *Lactobacilli* counts were measured by quantitative PCR following the method described in Maldonado-Lobón et al.,^14^ except for the common thermal profile applied for amplification, using this time 95°C for 5 minutes followed by 40 cycles of 95°C for 30 seconds and 65°C for 60 seconds, and a final melting curve from 55°C to 95°C.

For *L. fermentum* detection, breast milk samples (250–500 μL) were used as inocula for *Lactobacillus* spp. enrichment cultures performed in three different broth media (15 mL): M.R.S. (Oxoid) supplemented with vancomycin (100 μg/mL; Sigma-Aldrich); M.R.S. pH adjusted to 4.5 and Rogosa broth. After 24–48 hours of anoxic incubation at 37°C, 100 μL of liquid cultures was spread on the equivalent corresponding agar medium, except for cultures performed in M.R.S. pH 4.5, which were spread on LAMVAB agar medium. Petri dishes were incubated for 48 hours at 37°C under anoxic atmosphere (AnaeroGen, Oxoid). Colonies with *Lactobacillus* characteristic morphology were selected and identified by MALDI-TOF mass spectrometry (Bruker Biotyper). In brief, a small amount of biomass was transferred from selected colonies to a 96-spot plate, then 1 μL of matrix solution (2-cyano-3-(4-hydroxyphenyl) acrylic acid) was added over every sample and plate was dried at room temperature for 10–15 minutes. Analytical runs included *L. fermentum* CECT 5716 as positive control and accurate identification at species level was assumed when control and samples showed a score greater or equal to 2.2.

### Interleukin-8 quantification in breast milk

Interleukin (IL)-8 concentrations were measured in breast milk samples by ELISA quantification kits following the manufacturer's instructions (Bethyl, Montgomery, TX and Diaclone, France).

### Statistical analysis

For continuous outcomes, statistical tests for differences in the effect of treatment per visit were performed using parametric (*t* test, if normality assumption is met) and nonparametric (Mann–Whitney *U* and bootstrap confidence intervals, if normality assumption is not met) tests.

For the mother's primary outcome, diagnosis of mastitis, the total number of events from the study period was counted and the incidence rates were obtained. For categorical outcomes per visit, chi-square test for binary or categorical responses was used. Finally, a more robust and accurate analysis through statistical modeling was performed to determine the effect of the treatment along the study adjusted by relevant covariates gathered in the study.

The models applied to the data were Linear Mixed Models for continuous data when the residuals were normally distributed, Ordinal Mixed Models when the data recorded were related to categories of number of frequencies a day/week/month or any type of variable with increasing or decreasing order for the defined categories, and Logistic Mixed Models when the outcome to be analyzed was binary responses. A Poisson regression model was used to examine differences in the number of events observed of diagnosed mastitis.

The tests were performed at the two-sided 5% significance level, and 95% confidence intervals were obtained for the estimates.

The statistical software used to perform the analysis was SPSS version 19 and R version 3.1.

## Results

### Participants

Recruitment was started in August 2013 and ended in April 2015. The intervention was completed in July 2015.

**FIG. 1.**
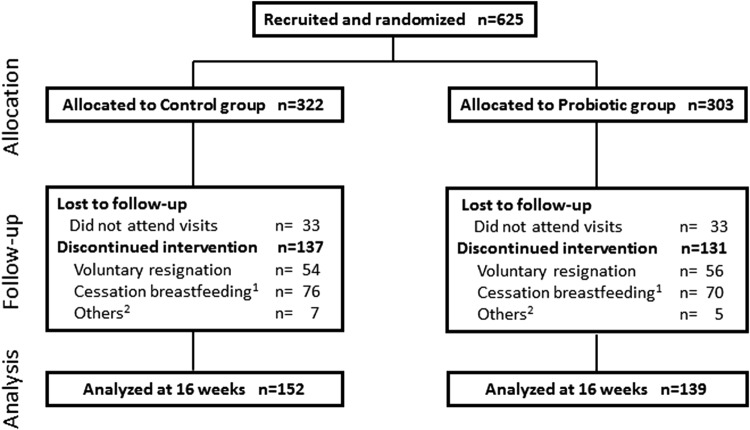
Flowchart of participants. (1) Causes: mother's decision, perception of insufficient milk, mastitis. (2) Causes: gastrointestinal problems in infants, maternal rash.

A flow chart of the participants in the study is shown in Figure 1. Six hundred twenty-five women were recruited for the study. Women were randomized and received the treatment (322 in control group and 303 in probiotic group). Three hundred thirty-four women discontinued the treatment because of causes reported in Figure 1. Four hundred twenty-five women completed 1 month of intervention (221 in control group and 203 in probiotic group), 359 completed 2 months of intervention (186 in control group and 173 in probiotic group), and 309 completed 3 months of intervention (165 in control group and 144 in probiotic group). Finally, 291 women (152 in the control group and 139 in probiotic group) finalized the intervention of 16 weeks. Data of these 291 women were included in the analysis of the mother's primary outcomes and incidence rates of mastitis during the first 4 months of breastfeeding. For evaluation of symptoms at each month, all data available were taken into account in the analysis.

No significant differences were detected between the baseline characteristics of women in the two groups (Table 1). Level of education and living area (city center, neighborhood, close to city, and rural area) of women were similar between groups (*p* = 0.446 and *p* = 0.216, respectively).

**Table 1.** Baseline Characteristics of the Participants of the Study

	*Control group (*n* = 322)*	*Probiotic group (*n* = 303)*	p*/chi-squared*
Mother's age (mean ± SD), years	32.19 ± 4.8	31.91 ± 4.9	0.487
C-section, *n* (%)	101 (31.4)	105 (34.7)	0.422
Multiparas, *n* (%)	133 (41.3)	113 (37.3)	0.777
Previous mastitis events in multiparas, *n* (%)	23 (17.3)	13 (11.5)	0.126
Use of pacifier, *n* (%)	98 (30.5)	94 (31.2)	0.862
Mixed breastfeeding, *n* (%)^a^	159 (49.4)	164 (54.3)	0.419
Sucking difficulties, *n* (%)	94 (29.2)	80 (26.4)	0.419
Number of daily breastfeedings, mean ± SD	9.03 ± 3.0	9.09 ± 2.6	0.655
Smoker at recruitment, *n* (%)	29 (9.0)	34 (11.2)	0.344

^a^ Number and percentage of infants who received infant formula as well as breast milk before starting the intervention.

Participants were asked to fill in a questionnaire regarding their diets at recruitment and at the end of the study period. Although some differences were detected between baseline and final time, no significant differences were detected between both groups (data not shown). Therefore, the significant effects observed for outcomes cannot be attributed to participant's different dietary habits.

### Incidence of mastitis

Sixteen women in the probiotic group suffered mastitis events in contrast to 30 in the control group. From events of diagnosed mastitis each month, the total number of events observed during the study for each group was built. A total of 58 events of mastitis were diagnosed in women of the study during intervention (incidence rate 0.199). Incidence rate of mastitis in probiotic group was significantly lower than that in the control group (IR = 0.130 in probiotic group versus IR = 0.263 in control group; *p* = 0.021) (Table 2). Therefore, the oral administration of *L. fermentum* CECT5716 during lactation decreased by 51% the incidence rate of clinical mastitis.

**Table 2.** Incidence of Mastitis During First 4 Months of Breastfeeding

	*Control group (*n* = 152)*			*Probiotic group (*n* = 139)*							
	*Events*	*IR*	*SE*	*Events*	*IR*	*SE*	*IRR*	*SE*	*IR decrease (%)*	*NNT*	p-*Value IRR*
Diagnosis of mastitis	40	0.263	0.042	18	0.130	0.031	2.018	1.328	50.6	8	0.021

*Women developed mastitis*	*Odd*	*Women developed mastitis*	*Odd*	*Odds ratio (probiotic/control)*	*NNT*	p
30	0.245	16	0.130	0.531	12	0.058

IR, incidence rate; IRR, incidence rate ratio; NNT, number of patients needed to treat; SE, standard error.

### Incidence of symptoms related to mastitis

Data about the incidence rate of different symptoms related to mastitis were also analyzed. The values of incidence rates of different symptoms were lower in the probiotic group (range 16–42% of decrease), reaching statistical significance for presence of heat zones in breast (*p* = 0.024). The most frequent symptom was breast pain. The distribution of the responses of breast pain during the first month of breastfeeding indicates higher percentage of women with breast pain in the control group than women in the probiotic group (43% versus 33%; *p* = 0.033). The odds of having breast pain in the probiotic group were significantly lower than odds of having breast pain in the control group (OR = 0.65; 95% CI 0.44–0.97). In particular, odds of breast pain in the control group are 1.5 times the odds of pain in the probiotic group. Percentage of women suffering from breast pain decreased with time, and in the fourth month only 6.5% of women in the probiotic group reported breast pain versus 9.2% in the control group (*p* = 0.515). Regarding intensity of breast pain, significant overall decrease along the study was observed in both groups. Although means of values of pain intensity were low, a slight difference between groups was detected in pain intensity during the first month of breastfeeding (2.92 in the control group versus 2.55 in the probiotic group; *p* = 0.05).

### Bacterial counts

Bacterial load was evaluated in milk samples of women at the end of the treatment and in case of mastitis event (Table 3). In the case of healthy women, lower level of *Staphylococcus* spp. was observed in breast milk of women of the probiotic group than in that of women in the control group (−48%; *p* = 0.013). In case of breast milk samples collected in case of mastitis events, lower level of *Staphylococcus* spp. load was also observed in samples from women of the probiotic group than in samples of women of the control group (−58%; *p* = 0.065). Significant differences were found in the *Streptococcus* and *Staphylococcus* loads, having lower values in average in women without mastitis. Higher level of *Staphylococcus* or *Streptococcus* is associated with the risk of suffering from mastitis [odds ratio for *Staphylococcus* is 1.961 (95% CI 1.272–3.025), *p* = 0.002; odds ratio for *Streptococcus* is 1.687 (95% CI 1.049–2.714), *p* = 0.031].

**Table 3.** Bacterial Counts and Concentration of Interleukin-8 in Breast Milk in Healthy Women at the End of Intervention (4 Months) and in Case of Mastitis Event

	*Group (control group,* n* = 123; probiotic group* n* = 126)*	*Healthy*	*95% Confidence interval (lower–upper level)*	p-*Value control versus probiotic*	*Mastitis*^a^*(control group,* n* = 49; probiotic group,* n* = 26)*	*95% Confidence interval (lower–upper level)*	p-*Value control versus probiotic*	p-*Value healthy versus mastitis*
*Staphylococcus*	Control	4.264 ± 0.72	4.100–4.420	0.013	4.642 ± 0.89	4.320–4.964	0.065	0.030
	Probiotic	3.983 ± 0.69	3.831–4.138		4.270 ± 0.57	4.025–4.515		0.080
*Streptococcus*	Control	4.948 ± 0.75	4.769–5.127	0.147	5.139 ± 0.803	4.867–5.411	0.647	0.114
	Probiotic	4.765 ± 0.65	4.616–4.929		5.501 ± 0.65	4.788–5.313		0.068
*Aerobes*	Control	4.314 ± 1.34	4.073–4.560	0.763	4.209 ± 1.19	3.763–4.654	0.488	0.637
	Probiotic	4.263 ± 1.25	4.051–4.508		3.996 ± 0.86	3.607–4.386		0.265
*Anaerobes*	Control	4.205 ± 1.25	3.994–4.434	0.326	4.266 ± 1.20	3.820–4.712	0.334	0.975
	Probiotic	4.050 ± 0.97	3.892–4.222		3.966 ± 0.89	3.560–4.372		0.789
*Lactobacillus*	Control	2.925 ± 0.68	2.717–3.191	0.486	2.976 ± 0.72	2.665–3.287	0.529	0.718
	Probiotic	3.050 ± 0.64	2.869–3.300		2.846 ± 0.17	2.746–2.946		0.202
IL-8	Control	1.84 ± 0.42	1.757–1.910	0.958	2.437 ± 0.46	2.177–2.697	0.037	<0.001
	Probiotic	1.84 ± 0.33	1.775–1.890		2.074 ± 0.68	1.870–2.279		0.034

Bacterial counts showed as a mean of log_10_ CFU/mL milk ± standard deviation. IL-8 showed as a mean of log_10_ pg/mL milk ± standard deviation.

^a^ Breast milk samples collected from mothers suffering from mastitis along the study.

IL, interleukin.

No significant differences were observed in *Lactobacillus*, total aerobes, and anaerobes counts.

Breast milk samples at the end of intervention were cultured in specific selective culture medium to favor the growth of *Lactobacillus* species. The specie *L. fermentum* was detected in breast milk in 23.6% of women in the probiotic group and in 14.6% of women in the control group.

### Inflammatory marker in breast milk (IL-8)

IL-8 concentration in milk was measured in samples of women at the end of the treatment and in case of mastitis event (Table 3). No significant differences were detected in IL-8 level between probiotic and control groups in the case of healthy women. However, IL-8 level was significantly higher in breast milk of women suffering from mastitis than in breast milk of healthy women. In these cases of mastitis events, women receiving probiotic strain showed significantly lower concentration of IL-8 in breast milk than that in breast milk in women of the control group (*p* = 0.037).

A general association of *Staphylococuss* with IL-8 was detected, which means that with per unit increase of bacterial load, the concentration of IL-8 increases significantly in 0.147 U (*p* < 0.001). The odds of having mastitis increase five times for unit increase of IL-8 [odds ratio = 5.502 (95% CI 2.904–10.425), *p* = 0.000].

### Pharmacological treatments

In the probiotic group, four women (2.9%) received antibiotic treatment in contrast to eight women (5.3%) in the control group; however, difference was not statistically significant (*p* = 0.308). Analgesic consumption was lower in the probiotic group, but differences between groups were not statistically significant. Just a significant difference between groups is detected during the third month of intervention when percentage of analgesic treatment related to breast symptoms is lower in the probiotic group [11 women in the control group corresponding to 6.7% versus no treatment reported in the probiotic group during this period (0%; *p* = 0.001)]. The use of topic treatments for nipple cracks (pomade with lanoline) was mostly observed in the first month of breastfeeding (4.7% of women used this kind of treatment). However the use of topical treatment was less frequent in the probiotic group [13 women in control group corresponding to 6% versus 4 women in probiotic group (2%; *p* = 0.048).].

## Discussion

*Staphylococcus* spp. is considered the most common etiological agent of mastitis.^6,9,10^ High counts of *Staphylococcus* spp. in breast milk are related to mastitis and painful breastfeeding.^14,21^ Previous studies have demonstrated the capability of *L. fermentum* CECT5716 to reduce *Staphylococcus* spp. load in breast milk of women suffering from mastitis and painful breastfeeding.^13,14^ The results of this study show that the consumption of *L. fermentum* CECT5716 during the breastfeeding period significantly reduces the incidence rate of mastitis by 51%. In the control group, 24.6% of women suffered from mastitis. Data about incidence of mastitis are very variable.^9^ This variability is probably because of differences in diagnosis criteria. Mastitis incidence reported by studies using the same diagnosis criteria of *L. fermentum* consumption was slightly lower, between 13% and 20%.^10,11,20^ It has been previously reported that antibiotherapy during delivery was significantly more widely administered to women reporting mastitis (OR 1.53).^22^ These data might explain the slightly higher incidence observed in the *L. fermentum* study because women who received preventive antibiotic in the context of delivery were recruited. Regarding efficacy of probiotic strains to prevent mastitis, Fernández et al.^11^ evaluated the efficacy of a probiotic strain, *Lactobacillus salivarius* PS2, to prevent mastitis. They observed a similar decrease of mastitis incidence during the first 3 months of breastfeeding (56%) for probiotic consumption. Although values of incidence reduction are similar to those of the *L. fermentum* study, there are important differences in protocols, apart from the different evaluated *Lactobacillus* species. For example, in the Fernández study, *L. salivarius* was administered during the last 10 weeks of pregnancy, but probiotic administration was not continued during breastfeeding. In the *L. fermentum* study, in which the probiotic strain was administered during breastfeeding, most episodes of mastitis occurred along the first 4 weeks postpartum. New studies should evaluate whether administration of *L. fermentum* during some weeks before childbirth might help to prevent more efficiently early episodes of mastitis.

Breast pain was quite frequent in women mainly during the first month, but in most cases it was not associated with clinical mastitis. *L. fermentum* consumption reduced the odds of suffering from pain and its intensity. In fact, in women who suffered from mastitis, the level of IL-8, a cytokine directly related to pain,^23^ was significantly lower in the probiotic group. Levels of IL-8 in breast milk were also directly related to *Staphylococcus* load in milk. This relation was also observed in a previous study performed in women suffering from painful breastfeeding.^14^ IL-8 is considered as an indicator of mastitis,^9,24^ so the reduction observed in the probiotic group in women suffering from mastitis is also indicative of lower level of inflammation and severity of mastitis.

Levels of *Staphylococcus* in milk were significantly lower in women receiving *L. fermentum* CECT5716. As high level of *Staphylococcus* is a risk factor for lactational mastitis, the reduction in this bacterial count might be responsible for the observed reduction in the incidence of mastitis. In fact, although higher level of *Staphylococcus* was found in milk of women who suffered from mastitis during the study, in the probiotic group this level tended to be lower.

The mechanism by which *L. fermentum* CECT5716 reduces the load of *Staphylococcus* in breast milk is not totally clear, although different activities of the probiotic strain might be involved. Antibacterial activity or competition phenomena have been described for *L. fermentum* CECT5716 in in vitro assays.^16^ However, although *L. fermentum* was detected in 61% more women in the probiotic group than women in the control group, we could not observe a significant presence of the strain in breast milk at the end of the intervention. Therefore, maybe local activity in the mammary gland is not mainly responsible for the activity. Other authors have suggested that metabolites with antibacterial properties produced by probiotic strains might reach the mammary gland and affect survival or virulence of *Staphylococcus*.^11^ *L. fermentum* showed in vitro the capability of inhibiting *Staphylococcus* growth in a diffusion agar assay, evidencing the production of antibacterial compounds that were not identified.^16^ In contrast, *L. fermentum* CECT5716 has shown immunoenhancing activity by increasing immunoglobulins and Th1 cytokines such as tumor necrosis factor (TNF)-α and IL-12.^18^ *Staphylococcus* spp. have a mechanism to avoid phagocytosis, the primary cellular defense of the mammary gland against pathogens.^25^ In animal models, *L. fermentum* CECT5716 increased the phagocytic activity of circulating blood leukocytes. This kind of activity in humans might help to prevent the proliferation of *Staphylococcus* spp. in the mammary gland.^17^

A limitation of this study is the high level of dropouts during the study. Although women with firm intention of breastfeeding during the first 4 months were recruited, around 25% of women stop breastfeeding during the intervention. In Spain, where this study was performed, data of National Institute of Statistic estimated the prevalence of breastfeeding at the third month of lactation to be 53.5%.^26^ Most of the dropout cases took place during the first month of study. Women reported that they were too busy with their babies to commit to the study. Despite the number of women who complete the study was significantly lower than planned, the study keeps enough statistical power. It is because of a higher incidence of mastitis finally observed and the high difference in the level of incidence between groups.

The other limitation of the study is the population. To observe higher level of incidence, only women receiving preventive antibiotic were included. In another recent study performed with the other probiotic strain, *L. salivarius* PS2, population with history of mastitis was selected.^11^ In this study, the decrease of mastitis incidence in the probiotic group was similar to that observed in our study. Therefore, although more studies should be performed in the general population, the capability of certain probiotic strains to protect against mastitis does not seem to depend on these risk factors.

Besides being a randomized double-blinded trial controlled by placebo, another strength of this study is the measurement in the same study of the incidence of a disease, its risk factors, and parameters indicative of the severity of the disease.

Exclusive breastfeeding is recommended up to 6 months of age by the WHO because breast milk provides all the nutrients and components necessary for proper infant development and growth.^27^ Mastitis is an important health problem that affects a high percentage of women during lactation. This problem has a direct impact not only on the mothers threatening their health but also on the infants, as mastitis makes the normal breastfeeding difficult and in some cases can even lead to abandonment of breastfeeding. Otherwise, the symptoms of mastitis affect the mother–infant relationship because the symptoms of pain associated with mastitis might cause anxiety and feeling of anger because of the pain problems during breastfeeding.^14^ Until now, an appropriate management of breastfeeding is the recommendation for mastitis prevention.^9^ Results of this study show that *L. fermentum* CECT5716 consumption might be a new and efficient strategy to prevent the development of lactational mastitis.
